# A global perspective of lung transplantation: Part 1 - Recipient selection and choice of procedure

**DOI:** 10.21542/gcsp.2016.5

**Published:** 2016-03-31

**Authors:** Reda E. Girgis, Asghar Khaghani

**Affiliations:** 1 Richard DeVos Heart and Lung Transplant Program, Spectrum Health,; 2 Michigan State University, College of Human Medicine, Grand Rapids, MI, USA

## Abstract

Lung transplantation has grown considerably in recent years and its availability has spread to an expanding number of countries worldwide. Importantly, survival has also steadily improved, making this an increasingly viable procedure for patients with end-stage lung disease and limited life expectancy. In this first of a series of articles, recipient selection and type of transplant operation are reviewed. Pulmonary fibrotic disorders are now the most indication in the U.S., followed by chronic obstructive pulmonary disease and cystic fibrosis. Transplant centers have liberalized criteria to include older and more critically ill candidates. A careful, systematic, multi-disciplinary selection process is critical in identifying potential barriers that may increase risk and optimize long-term outcomes.

## Introduction to the series

Lung transplantation (LTX) has witnessed dramatic growth worldwide in the past 15 years, making it an increasingly viable option for patients with refractory end-stage lung disease. Changes in allocation policies along with expansion of both donor and recipient selection criteria have contributed to the surge in volumes. Novel methods of ex-vivo lung perfusion have the potential to further expand the donor pool and widen availability of lung transplantation. Continued refinements in surgical techniques and peri-operative management have led to progressive improvements in early outcomes. Advancements in immunosuppressive and antimicrobial regimens have been accompanied by a reduction in acute rejection and infectious complications. While chronic lung allograft dysfunction remains the major limitation to long-term survival, ongoing efforts to better characterize this process and understand the pathogenesis will hopefully lead to breakthroughs in its prevention and treatment. This series of articles will provide an overview of the key aspects of lung transplantation with an emphasis on recent developments in the field.

**Figure 1.**
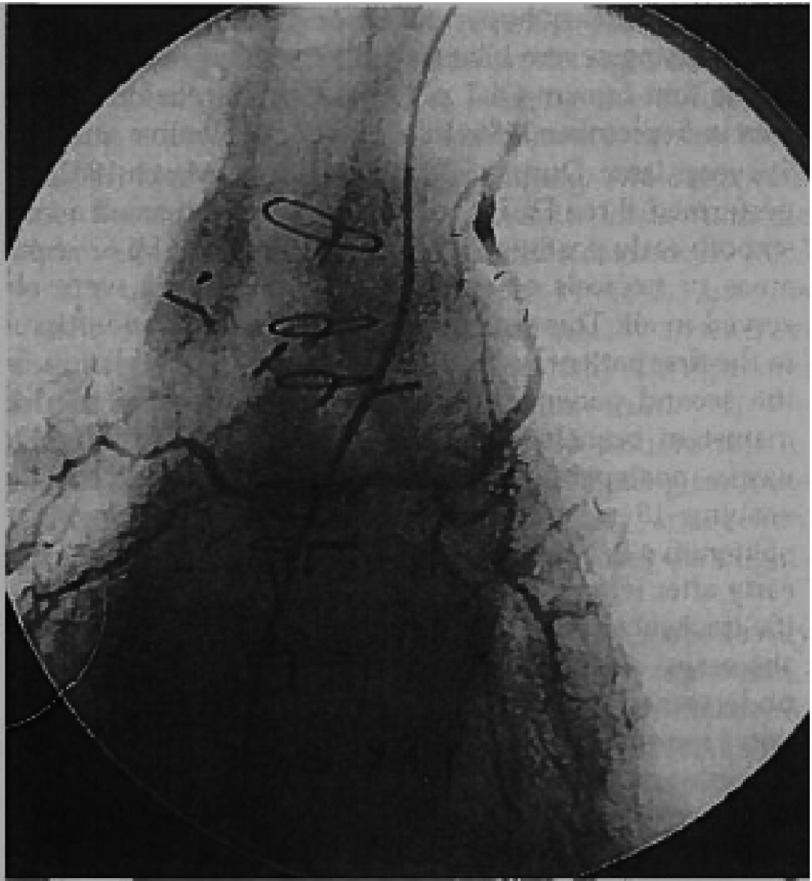
Bronchial arteriogram showing recipient left internal thoracic arteries perfusing both right and left bronchial arteries from a common trunk. (From reference #^5^ with permission.)

### Historical perspectives

After several failed attempts starting in the 1960’s, the first successful procedure was a combined heart-lung transplant by Dr. Bruce Reitz at Stanford in 1981^1^. The technique of single lung transplantation (SLTx) was developed by the Toronto group led by Dr. Joel Cooper, initially for pulmonary fibrotic disease, later that decade^2^. While SLTx was successfully applied to obstructive and pulmonary vascular disease^3,4^, this procedure is often not ideal for these indications, as discussed later and is unsuitable for suppurative lung disease. The first en-bloc double lung transplants were performed in 1986 by the Harefield Hospital team led by Sir Magdi Yacoub. However, these were associated with a high rate of tracheal anastomotic complications, a problem that was alleviated by the use of direct bronchial artery revascularization (Figure 1)^5^. Adequate airway healing was similarly achieved after bilateral sequential lung transplantation (BLTx) with bronchial anastomoses, which also allowed the avoidance of cardiopulmonary bypass. BLTx subsequently became the standard technique when both lungs required replacement^6^.

**Figure 2.**
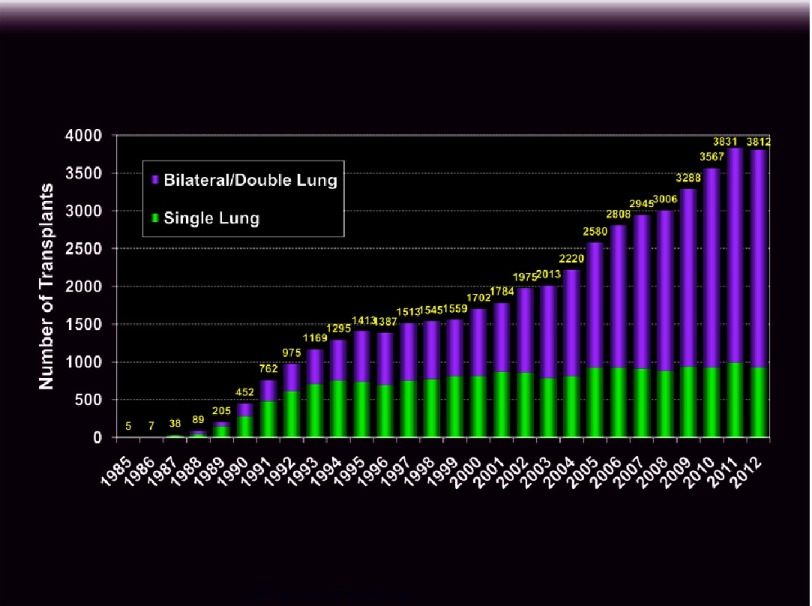
Number of adult and pediatric lung transplant reported to the International Society for Heart and Lung Transplantation by year (ishlt.org/registries/slides.asp?slides=heartLungRegistry, 2014).

In the early years, donors were transported to the recipient hospital where the surgeries were done in adjacent operating rooms. The introduction of distant organ procurement at Harefield^7^ allowed the rapid expansion of lung transplantation from 89 worldwide in 1989 to over 1400 in 1995^8^. Volumes continued to rise slowly until 2005. In that year, a major change in the donor allocation scheme in the U.S. was instituted. Prioritization of candidates based on severity of disease was also undertaken in Europe in the past decade^9–11^. These modifications, along with liberalization of both recipient and donor selection criteria led to a rapid acceleration in lung transplant volumes to nearly 4000 in 2011 (Figure 2)^8^. Further advancements in recipient support and donor management, as discussed below and in subsequent articles in this series, are expected to lead to a continued rise in the number of lung transplants, which will likely surpass heart transplants in the near future.

The availability of LTX has now expanded to several developing nations. Brazil^12^ and Argentina^13^ have had active centers for well over a decade. China recently reported their initial outcomes^14^ and India has embarked on a program^15^. In the Middle East, lung transplantation is available in Turkey^16^ and Iran^17^. Based on data reported to the International Society for Heart and Lung Transplantation (ISHLT) registry, 154 centers in 28 countries reported at least one lung transplant between 2011 and 2013 (ISHLT communication).

**Table 1.** Guidelines for Lung Transplantation for Common Disease Indications (adapted from ^18^).

**Idiopathic Pulmonary Fibrosis**	**COPD**
Timing of Referral: ⋅ At the time of clinical diagnosis Timing of Listing ⋅ FVC < 65% predicted ⋅ DLCO < 40% predicted ⋅ 6 MWD < 250 meters ⋅ ≥10% decline in FVC over 6 months ⋅ ≥15% decline in DLCO over 6 months ⋅> 50 m decline in 6 MWD over 6 m ⋅ Desaturation to < 88% during 6 MW ⋅ Extensive and/or worsening fibrosis on HRCT ⋅ Presence of significant pulmonary hypertension ⋅ Moderate to severe and/or worsening dyspnea ⋅ History of respiratory hospitalization	Timing of Referral (presence of 1 or more): ⋅ BODE index (composite score of body mass index, FEV_1_, degree of dyspnea and 6 minute walk distance) ≥ 5 ⋅ Hypoxemia (PaO_2_ < 60 mmHg) and/or hypercapnia (PaCO_2_ > 50 mmHg) ⋅ FEV_1_ < 25% of predicted ⋅ Progressive disease despite optimal medical therapy, including pulmonary rehabilitation Timing of listing (presence of 1 or more) ⋅ BODE index ≥ 7. ⋅ FEV_1_ < 15% to 20% predicted. ⋅ Frequent exacerbations ⋅ Episode of acute hypercapnic respiratory failure ⋅ Moderate to severe pulmonary hypertension.
**Cystic Fibrosis**	**Pulmonary Arterial Hypertension (PAH)**
Timing of Referral: ⋅ FEV_1_ < 30% of predicted, or a rapid decline in FEV_1_, particularly in females ⋅ Increasing frequency of exacerbations ⋅ Exacerbation requiring non-invasive ventilation ⋅ Recurrent or refractory pneumothorax or massive hemoptysis ⋅ Worsening nutritional status despite supplementation ⋅ Increasing antibiotic resistance ⋅ 6 minute walk < 400 m Timing of Transplant ⋅ Hypercapnia (PaCO_2_ > 50 mmHg) ⋅ Hypoxemia (PaO_2_ < 60 mmHg) ⋅ Advanced functional limitation ⋅ Pulmonary hypertension	Timing of Referral ⋅ NYHA functional class III–IV with escalating therapy ⋅ Rapidly progressive disease ⋅ Known or suspected pulmonary venoocclusive disease or pulmonary capillary hemangiomatosis ⋅ Timing of Transplant ⋅ Persistent NYHA class III-IV despite maximal medical therapy ⋅ Cardiac index < 2 L/min/m^2^⋅ Right atrial pressure > 15 mmHg ⋅ Other clinical and/or imaging evidence of RV failure ⋅ Massive hemoptysis

**Table 2.** Contraindications to Lung Transplantation.

⋅ Absolute Contraindications
1. Active smoking OR substance/narcotic abuse- must be abstinent for >6 months
2. Lack of adequate social support
3. Psychiatric or psychological condition associated with the inability to cooperate or comply with medical therapy.
4. Documented non-adherence or poor compliance with medical therapy and follow up care.
5. Malignancy within past 2 – 5 years, except cutaneous squamous and basal cell tumors.
6. Advanced, untreatable dysfunction of other major organ system:
a. Renal: creatinine clearance < 40 mg/min
b. Hepatic: cirrhosis and/or Child-Pugh class 2-3 functional impairment
c. Cardiac: Severe CAD not amenable to intervention, severe left ventricular dysfunction or valvular heart disease (except tricuspid regurgitation). Concomitant cardiac repair if feasible can be considered. Combined heart-lung if irreparable cardiac disease present can be considered.
d. Severe neurologic deficits that will impede rehabilitation potential
e. Other advanced or uncontrolled extra-pulmonary disease that is expected to severely impede rehabilitation potential or have a major impact on general health and survival, e.g. disabling arthritis, inflammatory bowel disease, bone marrow dysfunction etc.
7. Severe chest wall/spinal deformity, bilateral diaphragmatic paralysis.
8. Chronic active extrapulmonary infections
9. Cystic fibrosis patients colonized with B. cepacia, genomvar III
⋅ Relative Contraindications
1. Age > 65–70 yrs. Older patients can be evaluated on a case by case basis.
2. Obesity (BMI > 30). Patients with BMI 30–35 can be evaluated while attempts at weight loss are ongoing.
3. Severe debility, malnutrition/cachexia; non-ambulatory.
4. Active myositis or other chronic inflammatory disease requiring > 10 mg daily of prednisone to control
5. Connective tissue disease with significant extra-pulmonary involvement, e.g. vasculitis, nephropathy.
6. Respiratory muscle weakness
7. Severe or symptomatic osteoporosis
8. Chronic Mechanical ventilation. Can be considered if ambulatory and participating in rehabilitation.
9. Critical illness, e.g. shock, ECMO, acute respiratory failure.
10. Previous thoracic surgery. If unilateral, consider contralateral single lung. For prior CABG, right single preferred if possible.
11. Extensive pleural thickening
12. Colonization with highly resistant or highly virulent bacteria, fungi or mycobacteria
13. Severe upper GI dysfunction
a. Upper deglutition problems resulting in aspiration
b. Severe esophageal dysmotility
c. Gastroparesis
d. Severe GERD that will not be amenable to fundoplication
14. Other co-morbidities not leading to end-stage organ damage such as diabetes, HTN, should be optimally managed before transplant.

## Part 1: Recipient selection and choice of procedure

### Recipient selection

**Figure 3.**
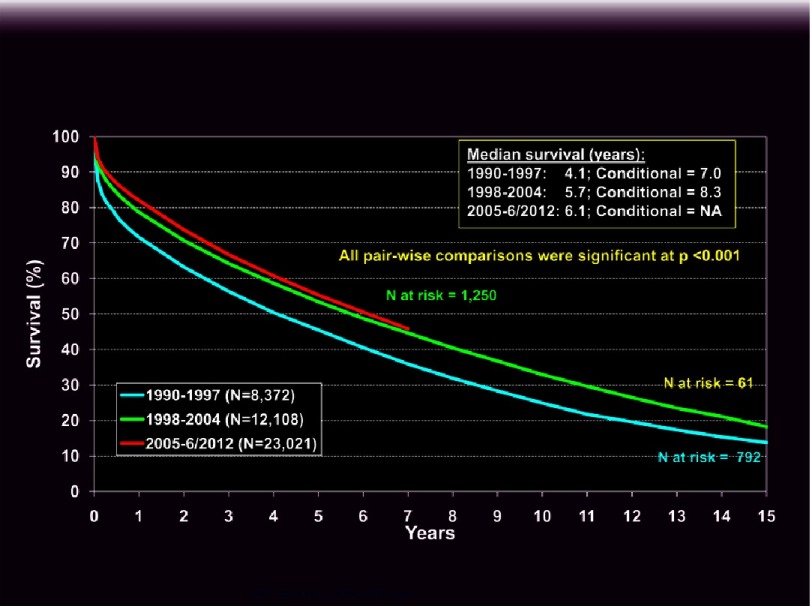
Kaplan-Meier survival of adult lung transplants by era as reported to the ISHLT (ishlt.org/registries/slides.asp?slides=heartLungRegistry, 2014).

Lung transplantation involves a complex surgical procedure and medical management process that can be quite challenging both physically and emotionally on the recipient and their care-givers. Even an uncomplicated lung transplant patient is not a “normal, healthy” individual and requires constant and careful monitoring by a multi-disciplinary team to enhance the prospects for a successful long-term outcome. Ideal candidates are free of co-morbidities, committed to the procedure and have strong psychosocial support. Given the potential risks, LTX candidates should have refractory end-stage lung disease with advanced functional limitations and a limited expected survival^18^. Median survival after LTX in the most recent era is 6.1 years (Figure 3)^8^. General guidelines for the most common disease indications are summarized in Table 1 and detailed below. Typical contraindications are listed in Table 2^18^ and discussed subsequently. A meticulous systematic evaluation is mandatory to assess the severity of the disease and detect potential risks for a poor outcome. The evaluation protocol used at our center is outlined in Box 1.

**Figure 4.**
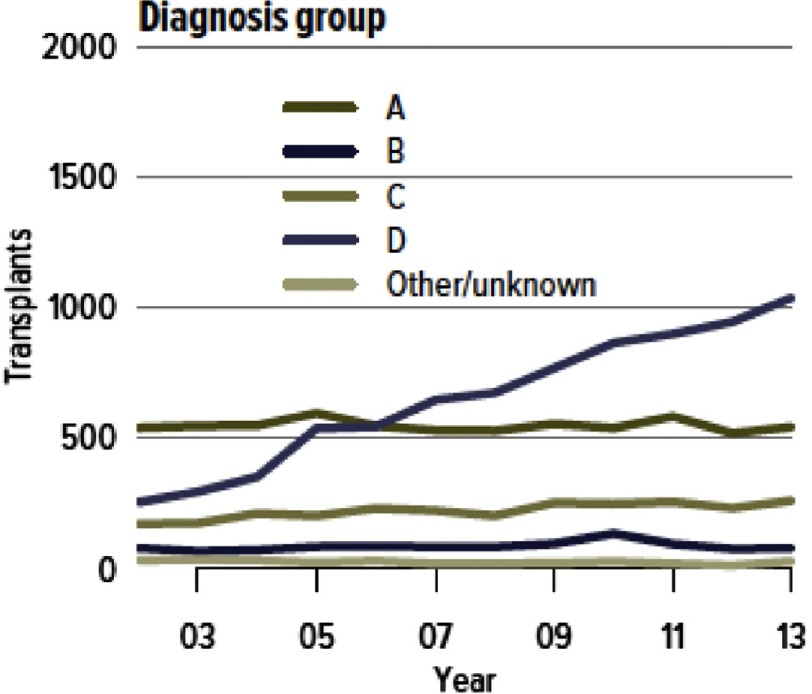
Number of lung transplants per year by diagnostic category in the United States. Group A: Obstructive Lung Disease; Group B: Pulmonary Vascular Disease; Group C: Cystic Fibrosis; Group D: Restrictive Lung Disease. (From reference # 19 with permission.)

### Interstitial lung disease (ILD)

Pulmonary fibrotic disorders are becoming the most common indication for LTX worldwide. The number of transplants performed for the predominant disease in this category, idiopathic pulmonary fibrosis (IPF), more than doubled in the ISHLT registry from 469 in 2004 to 1012 recipients in 2011, approaching the 1167 cases done for COPD/emphysema^8^. In the U.S., pulmonary fibrosis surpassed COPD as the leading diagnosis category in 2007 and accounted for more than half of all transplants done in 2013 (Figure 4)^19^. As described later, this pattern is largely the result of the introduction of lung allocation schemes which give high priority to this diagnosis given its high waitlist mortality. In addition, there is data suggesting that the incidence of IPF is increasing. A U.K. study reported a doubling of the incidence from 1990 to 2003^20^.

Recent years have witnessed considerable progress towards understanding the pathogenesis of IPF^21^, including identification of several genetic factors^22,23^. In May of 2014, clinical trial results of pirfenidone^24^ and nintedanib^25^ were reported. Both agents significantly reduced the decline in forced vital capacity and represent the first effective medical therapies for IPF, after numerous other failed agents. Nevertheless, these drugs appear to only slow the progression of disease, which can often be rapid. Therefore, potential candidates should be referred to a transplant center once the diagnosis is established and listed once any sign of deterioration is detected^26^. Early referral allows time for identification and intervention for potential medical and psychosocial barriers to transplant and for patient and caregiver education.

BOX 1 Components of Lung Transplant Evaluation

1. Complete history and physical, including detailed family history
2. Consultations
  i. Transplant Surgery
  ii. Psychosocial evaluation
  iii. Nutrition
  iv. Transplant infectious disease
  v. Transplant pharmacy
  vi. Financial coordinator
  vii. Transplant coordinator: This visit will include a detailed orientation of the pre and post lung transplant process, including post-operative risks, management, outcomes etc.
  viii. Contraceptive counselling for women of child-bearing potential.
  ix. Other consultations as dictated by clinical scenario, e.g. rheumatology for connective tissue disease assessment.
3. Pulmonary Evaluation
  i. PA and lateral chest x-ray
  ii. Spiral CT of chest with contrast
  iii. Pulmonary Function test with DLCO
  iv. Arterial blood gases on room air
  v. 6 minute walk distance with pre and post saturation
  vi. V/Q scan with differential quantitation for single lung transplant cases
  vii. Diaphragmatic sniff test: ultrasound or fluoroscopy
  viii. Quantiferon test/T-Spot test
4. Cardiovascular Evaluation
  i. EKG
  ii. Echocardiogram
  iii. Right heart catheterization
  iv. Left heart catheterization with coronary angiography for patient age >50 or >45 and strong family history or clinical suspicion.
  v. Consider stress testing (dobutamine echo) if known CAD
  vi. Carotid Doppler for high risk patients >60 y, h/o neurologic event, and patients found to have CAD
  vii. Lower extremity ABI for high risk >60 y, history or exam suggestive of PVD, diabetes, and patients found to have CAD.
5. GI Evaluation
  i. Spiral CT of abdomen and pelvis with contrast
  ii. Colonoscopy or CT colonography for age >50 y
  iii. Cine-esophagogram on all patients
    1. If reflux and/or esophageal dysmotility, consider 24 hr pH/impedance probe and esophageal manometry
    2. Consider gastric emptying study
    3. Consider GI consultation
6. Other Testing
  i. 24 hour urine for creatinine clearance
    1. If the calculated GFR is <40
  ii. Bone mineral density (DXA scan)
  iii. Mammogram for women >40
  iv. PAP smear annually
  v. Dental clearance
7. Laboratory tests
  i. Full chemistry panel, CBC with differential, Uric acid, Lipid panel, Thyroid function, Iron, TIBC, ferritin, Vitamin D level, PT/PTT, Pre-albumin, Hgb A1C
  ii. Urine drug screen
  iii. Blood cotinine level
  iv. Urinanalyis, with micro
  v. Urine albumin to creatinine ratio if diabetic
  vi. Hypercouaguble work up if there is a personal or family history of venous and/or arterial thrombosis
  vii. Stool for occult blood if no colonoscopy
  viii. PSA in males >40 or younger if family history of prostate cancer
  ix. Sputum gram stain and culture (routine, fungal and AFB) if productive cough present and in all CF/bronchiectasis patients.
  x. MRSA nasal swab
  xi. Anti-HLA antibodies (PRA)
  xii. Blood type, Tissue typing (prior to listing)
  xiii. Serology: CMV IgG, HIV, viral hepatitis, EBV, RPR, HSV, VZV, MMR, toxoplasmosis

### Choice of transplant procedure for ILD

**Figure 5.**
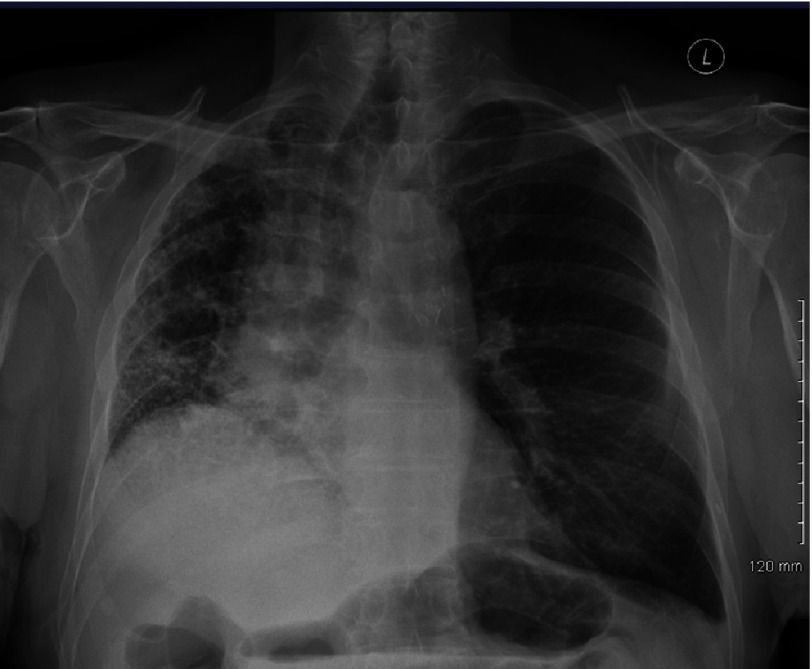
Chest radiograph of a 71 year old recipient 5 months after left single lung transplant for IPF. The allograft is well expanded with mediastinal shift towards the native lung. The forced vital capacity was 72% of predicted and FEV_1_ was 87%.

**Figure 6.**
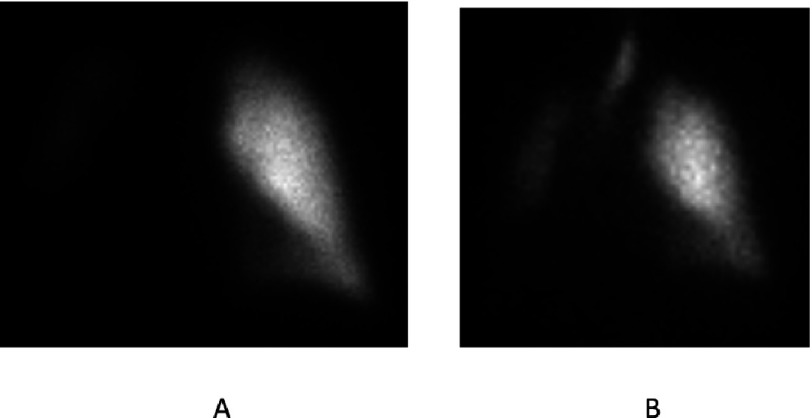
Perfusion (A) and ventilation (B) radionucleotide scan images of patient from Figure 5. The left lung allograft accounts for 95% of perfusion and 97% of ventilation.

Either single (SLTx) or bilateral lung transplants (BLTx) are suitable for IPF and other ILD’s. In the past, the majority (80% in 1998) were single, but in recent years, about half have been bilateral procedures^8^. From a physiologic perspective, SLT is ideally suited for restrictive lung disease where the native lung is stiff, allowing ample expansion of the allograft with normal compliance (Figure 5). The vital capacity typically exceeds 70% of predicted 9-12 months post-transplant in uncomplicated cases and the majority of both ventilation and perfusion are directed to the allograft soon after surgery (Figure 6). While lung volumes and diffusing capacity for carbon monoxide (DLCO) are greater after BLTx, exercise capacity, which is primarily limited by peripheral muscle factors, is no different^27^. Quality of life is also similar^28,29^ and in fact bodily pain may be greater after bilateral transplant^30^.

**Figure 7.**
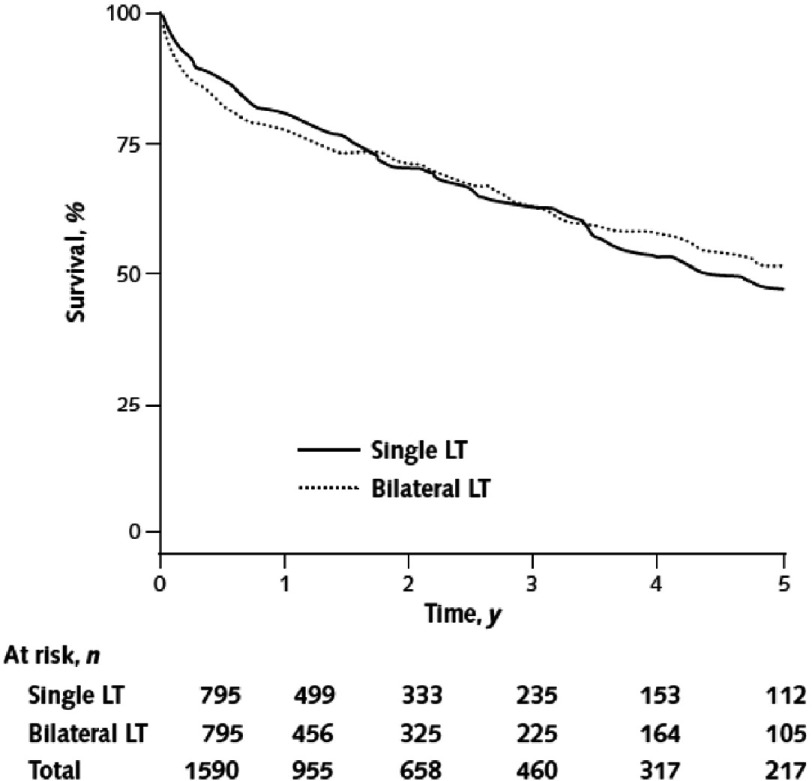
Survival after single or bilateral lung transplant for Idiopathic Pulmonary Fibrosis in 795 propensity matched pairs. (From reference #^32^ with permission.)

The shift towards BLTx has largely been driven by the finding of better long-term survival. Data from the ISHLT registry from 1990 to 2011 shows comparable one year outcomes, after which the survival curves diverge in favor of BLTx with 5 yr survival of 52.8% vs. 43.3% for SLTx^31^. Importantly, this data is not controlled for other factors which could impact survival. BLTx is more likely to be offered to younger, healthier subjects with less comorbidity. Indeed, an analysis of over 3000 transplants for IPF done in the U.S. between 1987 and 2009 found that SLTx recipients were significantly older (mean 57 vs. 54 years) with 39 % being >60 years compared with only 30% of those who received BLTx^32^. Median survival was 5.2 years after BLTx compared with 3.8 for SLTx. However, no significant difference was detected when only transplants done after 2001 were analyzed. Moreover, after adjusting for covariates, primarily recipient age and transplant year, survival was similar (Figure 7). There appeared to be an increased risk of early mortality in the first post-operative year with BLTx, and lower risk thereafter (hazard ratio of 1.18 and 0.72, respectively). BLTx recipients were more likely to die of primary graft failure, whereas death due to cancer (presumably in the native lung) was more common among the SLTx group where it accounted for over 12% of deaths. The proportions of deaths due to other causes, including chronic rejection, were comparable^32^. In another study using the same dataset, long-term survival adjusted for covariates, conditional upon one-year survival, was improved with BLTx (HR 0.73)^33^. Recipient age over 57 years was found to significantly increase one year mortality, but again, there was no difference in overall survival in any age group. Thus, there is no clear advantage to the routine use of BLTx for IPF. A notable exception is the presence of bronchiectasis or cavitary disease with chronic bacterial or fungal colonization that could contaminate the allograft in the case of a single LTX. In addition, the presence of very small thoracic cavities on both sides requiring use of relatively small donor lungs may favor the use of BLTx to provide greater ventilatory capacity.

Pulmonary hypertension is often cited as a rationale to perform BLTx, as pulmonary vascular disease in the native lung will force nearly all the cardiac output through the allograft. Elevated mean pulmonary artery pressure (mPAP) was associated with higher short-term mortality with either type of procedure in an analysis of ISHLT registry data between 1995 and 2002^34^. In the UNOS study of Thabut et al, BLTx was not protective, regardless of mPAP^32^. On the other hand, an analysis of IPF transplants done in the U.S. during the 31 months following institution of the Lung Allocation System (LAS) in 2005 suggested a significant 1 yr survival benefit (adjusted HR: 0.48) for BLTx among the highest LAS quartile (≥ 52)^35^. Interestingly, SLTx was associated with better 1 yr survival in the lowest LAS quartile. As described in a subsequent article, the LAS includes multiple measures of disease severity including PAP^36^. A recent multi-center study found that SLTx was an independent risk factor for severe primary graft dysfunction (PGD), the leading cause of early mortality following LTX^37^.

Ultimately, the decision to perform a single vs. bilateral procedure for ILD will depend on the preference of the surgeon and transplant center. The small disadvantage in early mortality with BLTx maybe attenuated with optimization of perioperative management, allowing the recipient to profit from the long-term survival benefit. From a societal standpoint, SLTx allows more patients to be transplanted and potentially greater number of total years of life saved. For the individual, waitlist time was longer and mortality greater for IPF candidates who were listed for only BLTx compared to those listed for SLTx or either in the U.S. UNOS database^38^. Similarly, an analysis in the U.K. concluded that accepting a single lung offer reduced mortality compared with waiting for a pair of organs^39^. In both studies, the findings for IPF did not apply to COPD, where no differences in survival between the two strategies were detected.

### Chronic obstructive pulmonary disease (COPD)

COPD is projected to become the third leading cause of death worldwide by the year 2020. While the proportion of transplants done for ILD continues to grow, COPD remains the leading indication internationally^31^. The decision regarding timing of transplant is often fraught with uncertainties. Patients with seemingly very advanced disease can remain stable for quite some time. In the U.S., COPD subjects on the transplant waitlist have a mortality rate of around 6 per 100 patient years, considerably lower than other diagnostic groups^40^. Consequently, it has been difficult to demonstrate a clear survival advantage with LTX. An analysis of UNOS data estimated that 45% of COPD recipients gained 1 year or more of life with transplant while 26% lost 1 year of life and the remainder would gain or lose less than 1 year^41^. Independent variables associated with mortality on the waitlist included age, functional status, oxygen requirement, six-minute walk distance, continuous mechanical ventilation, FEV_1_, systolic pulmonary artery pressure and body mass index. Alpha-1 antitrypsin deficiency candidates had better waitlist survival. Parameters linked with post-transplant mortality were age, continuous mechanical ventilation, functional status, presence of diabetes and type of procedure (bilateral better than single). Guidelines for referral and listing are outlined in table 1. Quality of life (QOL) considerations are also important for many COPD candidates aside from potential survival benefits.

For appropriate candidates, surgical lung volume reduction surgery (LVRS) should also be considered as an alternative to lung transplantation. This would comprise those with heterogenous, upper-lobe predominant emphysema, reduced exercise capacity despite pulmonary rehabilitation and an FEV_1_ or DLCO > 20% or predicted^42^. Prognostic factors in emphysema patients in the medical arm of the National Emphysema Treatment Trial (NETT) of LVRS included age, a modified BODE index, higher residual volume and greater lower lobe vs. upper lobe emphysema^43^. The performance of surgical LVRS does not preclude subsequent lung transplantation if response is inadequate or temporary^44^. However, it does appear that post-transplant morbidity and possibly mortality are higher, particularly for those who derive no significant improvement in lung function following LVRS^45^. The Pittsburgh group reported a prolonged ICU length of stay with increased incidence of bleeding requiring re-exploration, phrenic nerve injury and acute renal failure. Functional outcomes were also inferior and a trend for reduced early survival was noted^45^. Risk factors for mortality in this group were age >65, severe pulmonary hypertension (PASP > 60 mmHg), prolonged cardiopulmonary bypass (CPB) and high transfusion requirements. A variety of experimental bronchoscopic lung volume reduction techniques are currently being evaluated and consideration should be given to enrollment in one of these studies for patients with less advanced disease^46^.

**Figure 8.**
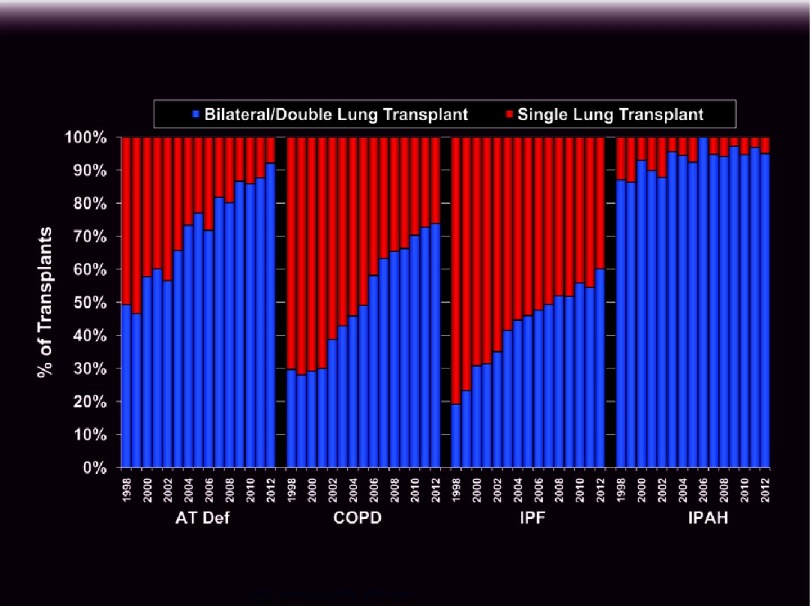
Distribution of single and bilateral lung transplants for different indications by year as reported to the ISHLT. AT Def: alpha-1 anti-trypsin deficiency (ishlt.org/registries/slides.asp?slides=heartLungRegistry, 2014).

#### Choice of transplant procedure for COPD

As with ILD, SLTx had been the preferred procedure for COPD in the past. In the last decade, however, an even more dramatic rise in the proportion of bilateral vs. single transplants has occurred in this group (Figure 8)^31^. In contrast to restrictive lung diseases, the native, more compliant emphysematous lung typically shifts across the mediastinum limiting expansion of the allograft. Vital capacity is on average much lower after SLTx for obstructive vs. restrictive lung disease, but again, exercise capacity and functional status are comparable and not ventilatory limited^27^. Occasionally, severe native lung hyperinflation with significant impairment in allograft function occurs, requiring lung volume reduction^47^. In the immediate post-operative period, allograft dysfunction can lead to life-threatening native lung hyperinflation requiring independent lung ventilation. Long term complications of SLTx include bronchogenic carcinoma of the native lung and a higher incidence of bronchiolitis obliterans syndrome (BOS), likely as a result of lower functional reserve^48^. A clear benefit in long-term survival of BLTx vs. SLTx for COPD has been observed (58% vs. 49% at 5 years)^31^. In contrast to ILD, this survival advantage persists after adjusting for baseline variables with a hazard ratio of 0.83 to 0.89^49^. However, no clear benefit was observed for recipients ≥ 60 years of age. Based on modeling, some authors have argued that in most circumstances, a policy of SLTx for COPD improves access to transplant for a larger number of candidates without significantly affecting post-transplant survival^50^. Nevertheless, a strong argument can be made in favor of a bilateral procedure in most individuals and roughly 90% of cases in the U.S. now undergo BLTx^40^.

**Figure 9.**
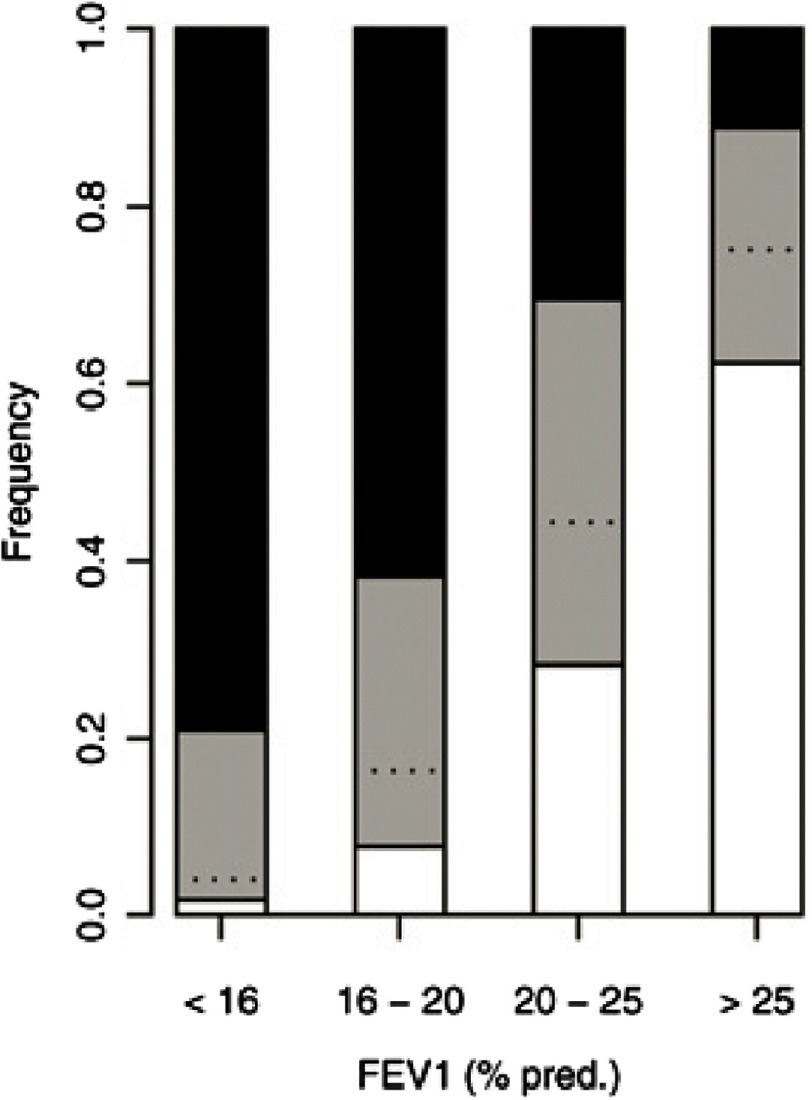
Survival effect of bilateral lung transplant in COPD according to quartiles of FEV_1_% predicted. Black areas of bars represent difference between expected median survival with and without transplant of > 1 yr (gain of life), white areas a loss of > 1 yr and shaded areas gain or loss of < 1 yr. Dashed lines separate between gain and loss of survival (taken from reference # 41 with permission).

### Cystic fibrosis (CF) and bronchiectasis

Advances in therapy and a comprehensive approach to the care of CF has resulted in a marked increase in life expectancy, which now approaches a median of 40 years^51^. Nevertheless, progressive pulmonary disease ultimately results in respiratory failure in most patients and CF consistently accounts for about 16% of all transplants^8^. Outcomes after lung transplantation for CF are superior to all other diagnostic groups given the younger age and absence of major co-morbidities. One and 5-year survival in the most recent era are 86% and 63%, respectively^8^. Guidelines for referral and transplant are listed in Table 1. Prognosis can be difficult to ascertain. While an FEV_1_ < 30% of predicted had excellent negative predictive value (97%) for predicting 2-yr mortality in the large CF Foundation database, the positive predictive value was only 28%^52^. Independent risk factors for waitlist mortality among 343 patients at 4 transplant centers were FEV_1_ < 30%, PaCO_2_ > 50 mmHg and use of a nutritional intervention, but not BMI^53^. In this study, 13% of patients who died on the waitlist had an FEV_1_ > 30%. Thus, this should not be used as the sole criterion to consider a CF patient for transplant. Referral from an accredited CF care center was associated with improved survival. Pulmonary hypertension (mPAP > 25 mmHg) by right heart catheterization was a strong predictor of waitlist mortality in the UNOS database and severe elevation in PAP (> 35 mmHg) was associated with as high as a 4-fold risk^54^. The impact of PH on waitlist mortality in CF was found to be nearly 2-fold greater than that observed with other diagnostic groups. Younger age and higher BMI were linked with better survival^54^. CF-related diabetes and *Burkolderia cepacia* infection are also associated with reduced survival^55^.

Ideally, recipients should be ill enough to warrant the risk of transplant, but well enough to survive until an organ is offered and the transplant procedure is viable. An analysis of survival benefit using the UNOS registry since implementation of the LAS demonstrated a 69% reduction in the risk of death^56^. One year mortality on the waitlist was 13%. While the benefit was greater with increasing LAS, even patients with scores of 31-35 appeared to demonstrate improved survival with transplant. Such patients would have preserved gas exchange and no pulmonary hypertension.

No formal guidelines exist for timing of transplantation in non-CF bronchiectasis. Prognostic factors include FEV_1_ < 30% predicted, BMI < 18.5, prior hospitalization and three or more exacerbations in the preceding year. A multi-dimensional bronchiectasis severity score has been developed that accurately predicts 4 year survival^57^. Suppurative lung disease requires a bilateral transplant procedure to avoid contamination of the allograft from the native lung.

#### Microbial considerations in CF

Chronic infection with *Burkholderia cepacia* complex has long been recognized to have a strong negative impact on survival in CF, imparting the equivalent of a 48% reduction in FEV_1_^58^. Unfortunately, such infections are extremely resistant to antibiotics and dramatically increase post-transplant mortality risk. With refinements in microbiologic classification, it has become apparent that infections with *B. cenocepacia* (genomvar III) accounts for most of the post-transplant deaths. A large study using the CF Foundation database further identified certain strains of *B. cenocepacia* that conferred the increased risk, along with *B. gladioli*, a non-cepacia complex species^59^. Most centers would consider infection with these organisms to be contraindications to transplant. No difference in outcome was observed with *B. multivorans* infection^59^. Pan-resistant *Pseudomonas aeruginosa* may be associated with a slightly increased risk, but not sufficient to preclude successful transplantation^60,61^. The impact of methicillin-resistant *Staph aureus* (MRSA) infection on post-transplant outcomes has not been reported. Non-tuberculous mycobacterial (NTM) infections are common in CF and should be routinely screened for in transplant candidates. *M. abscessus* can be quite resistant to therapy and represents a relative contraindication to transplant^62^. Colonization with Aspergillus species is common and increases the risk of post-transplant invasive disease, but not mortality^63^.

### Idiopathic pulmonary arterial hypertension (IPAH)

The last two decades have witnessed dramatic progress in the medical therapy of IPAH with 11 FDA approved agents from four pharmacologic classes currently available^64^. Despite aggressive treatment, however, morbidity and mortality remain high with 3-yr survival of roughly 60% in modern series^65,66^. Death is often due to progressive right heart failure. Symptoms, signs and hemodynamic evidence of right ventricular decompensation despite therapy portend a poor prognosis. Guidelines for transplantation are listed in Table 1.

While the proportion of lung transplants performed for IPAH has fallen considerably since 1990 from 12% to less than 3%, the actual number of cases has slowly grown, but remains quite small at around 100 per year worldwide^31^. IPAH is a relatively rare disease, but the small number of transplants performed while mortality remains high suggests that the procedure is underutilized for this diagnosis. Medical therapy clearly has had an impact on the disease with a reduction in mortality^67^. However, their efficacy is often limited and despite a transient stabilization or improvement in symptoms, reassuring the clinician, RV dysfunction can progress. Referral to lung transplant is thus often delayed.

A reluctance to refer PAH patients for transplant also likely reflects the historically poor outcomes. One year survival is the lowest among all diagnostic groups at 71% in the ISHLT registry since 1990^31^, largely due to high early post-operative complications including bleeding and primary graft dysfunction^68^. However, early outcomes continue to improve over time for all diagnostic groups and long-term survival conditional upon one-year survival for IPAH is among the highest (5-yr survival of 72% compared with 63% for IPF and COPD)^31^.

A bilateral or heart-lung transplant is required for PAH. Single lung transplantation is associated with an unacceptably high rate of early mortality due to the inability of the native lung to accommodate any significant portion of the cardiac output with consequently a high incidence of primary graft dysfunction and gas exchange derangements from ventilation-perfusion imbalance due to ongoing and equal ventilation of the native lung^27^. BLTx constitutes the majority of procedures performed for this indication both in the U.S.^69^ and worldwide^70^. Even with advanced right heart failure, cardiac function rapidly recovers once the pulmonary artery pressure is normalized^71^ obviating the need for a combined heart-lung procedure. Nevertheless, some centers prefer HLTx over BLTx in the setting of severe right ventricular dysfunction^72^. In contrast, PAH associated with congenital heart disease is the leading indication for HLTx. Simple cardiac defects, such as atrial and ventricular septal defects can be managed with repair at the time of BLTx^73^.

**Figure 10.**
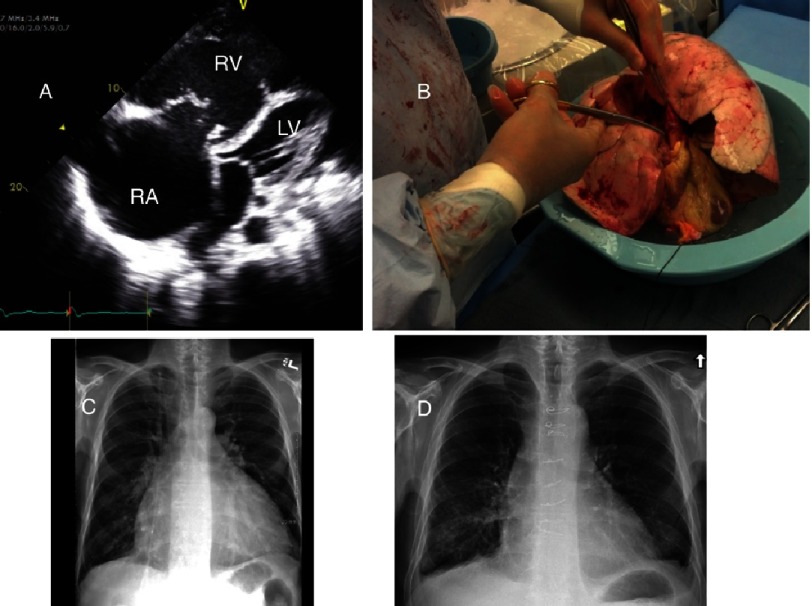
55 year-old male with Idiopathic Pulmonary Arterial Hypertension and severe refractory right heart failure despite therapy with intravenous epoprostenol, requiring continuous ambulatory dopamine infusion for inotropic support. Apical 4-chamber echocardiographic view (A) demonstrates massive dilatation of the right atrium (RA) and right ventricle (RV) impairing left ventricular (LV) filling. The patient underwent a combined heart-lung transplant (B) and is asymptomatic with normal cardiac and pulmonary function 2 years post-transplant. Chest radiograph pre (C) and post (D) transplant.

IPAH, as well as elevated pulmonary artery pressure, are well established risk factors for severe PGD^74^, which was reported in over one quarter of IPAH recipients in the UNOS/ISHLT registry^75^. The basis for this high incidence is not clear. One potential mechanism is sudden unloading of the right ventricle with consequent shear stress injury to the pulmonary vascular endothelium. Universal requirement for cardiopulmonary bypass and frequent blood product transfusions likely also play a role in the development of acute lung injury. Elevations in left atrial pressure from transient left ventricular dysfunction have been reported^76^, presumably a consequence of chronic under-filling of the left heart. A combined heart-lung transplant would eliminate the consequences of right heart pressure overload and left heart underfilling. However, the transplanted heart undergoes cold storage and there is often transient dysfunction due to ischemia-reperfusion injury. Bleeding can also be more problematic with the heart-lung procedure. There is no difference in survival between the two operations. However, patients are not randomized and heart-lung transplantation is often selected for those cases with the most advanced right heart failure (Figure 10)^72,76^.

### Other lung diseases

Pulmonary sarcoidosis accounts for 2-3% of all lung transplants^31^. Indications for transplant include advanced functional impairment, chronic respiratory failure and pulmonary hypertension^77^. Marked upper lobe fibrosis and pleural thickening can increase the risk for post-operative bleeding. Just over 1% of transplants are performed for lymphanigioleimyomatosis (LAM). A bilateral procedure avoids the potential complications of chylo-, or pneumothorax arising in the native lung. Large renal angiomyolipomas are at risk for bleeding and should be interrogated for before transplant^78^.

Pulmonary involvement in scleroderma or systemic sclerosis (SSc) is the most common cause of death in this disorder. The approach to scleroderma lung disease (ILD and/or PAH) varies considerably among transplant centers. Many programs routinely consider scleroderma a contraindication because of concern for esophageal dysmotility and risk of aspiration. Only 1.4% of transplants are for connective tissue diseases^31^. However, several studies have failed to show a difference in outcomes compared with IPF or IPAH^79^. In one series, there was no impact of esophageal dysfunction on outcomes, which were similar to non-scleroderma related ILD^80^. Nevertheless, it is not clear if any severity of esophageal disease is acceptable. Other co-morbidities also need to be carefully considered^81^.

Retransplantation, which accounts for 4% of all transplants^31^, is a viable option for recipients with advanced chronic allograft dysfunction without significant co-morbidity. Outcomes for such patients have improved in the modern era. However, repeat transplants done early post-operatively for primary graft dysfunction are associated with a high mortality rate^82^.

**Figure 11.**
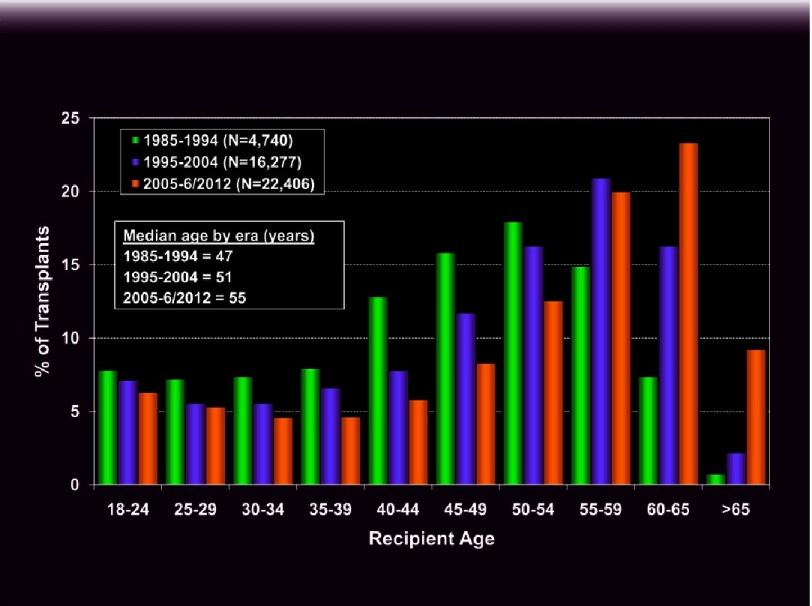
Lung transplant recipient age distribution by era (ishlt.org/registries/slides.asp?slides=heartLungRegistry, 2013).

### Special considerations in recipient selection

#### Age

There has been a dramatic shift towards transplantation of older individuals in recent years. Median recipient age in the ISHLT registry has increased from 47 yrs. two decades ago, to 55 yrs. between 2005–2012^8^ (Figure 11). This pattern has accelerated in recent years, particularly in the U.S. where 26% of LTX recipients were aged 65 or over in 2012, compared with just 7% in 2004^40^. Aging of the population and relaxation of recipient selection criteria account, in part, for this trend. However, the most important factor has been current allocation schemes (to be discussed in a separate article) that give considerable priority to pulmonary fibrosis patients, who are typically older. While short term outcomes are acceptable, recipient age is an independent risk factor for 1 yr. mortality, increasing exponentially after age 55 with a hazard ratio of 1.44 at 65 yrs^8^. Early survival has improved in recent years since introduction of the LAS with comparable 1 yr. survival of recipients aged ≥70 vs. those 60–69^83,84^. Of greater concern are the clearly inferior longer term results. Median survival was only 3.6 yrs. for those > 65 yrs. in the most recent ISHLT registry report. Reduced long-term survival in the elderly remains apparent even when conditional upon 90 d or 1 yr. survival^8^. A recent analysis of UNOS data of transplants after 2005 also demonstrates considerably lower survival at 3 and 5 years for recipients aged ≥ 70 vs. those aged 60-69^85^. Given the limited donor pool, offering this scarce resource to older subjects generates important ethical questions^86^. Age needs to be considered on a case-by-case basis and in conjunction with other factors when assessing a recipient’s candidacy. Co-morbidities often associated with ageing, such as diabetes, coronary artery disease and hypertension will further increase risk. The concept of frailty, which has been linked with morbidity and mortality in the elderly and correlated with general surgical outcomes^87^, may be a useful assessment in LTX, but remains to be investigated^88^. All candidates, regardless of age, should undergo physical rehabilitation to optimize their strength and conditioning prior to listing. Even the most debilitated patients can undergo an exercise therapy program and derive considerable benefit, both in terms of functional capacity and quality of life^89^.

**Figure 12.**
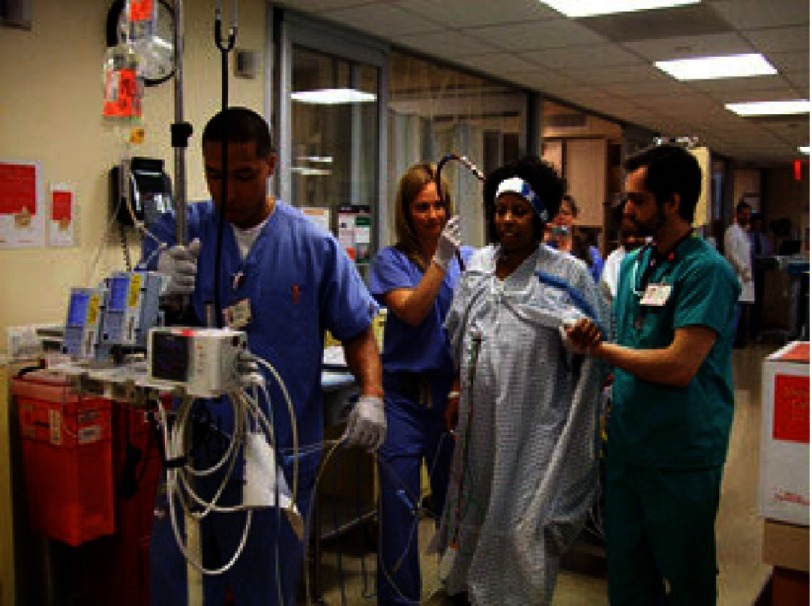
A non-ventilated patient ambulating with veno-venous ECMO while awaiting lung transplantation (http://vesta.cumc.columbia.edu/surgery/residency/Applicants/Research/ Cardiothoracic.php; accessed 10/10/15).

#### Body habitus and nutritional status

Obesity may interfere with lung expansion and limit rehabilitation. Two analyses of the UNOS database demonstrated a modestly increased mortality risk of 16-22% associated with BMI > 30 kg/m^2^^90,91^. The effect was observed for both restrictive and obstructive lung disease. Short-term outcomes appeared to be primarily impacted, as 5-year survival conditional upon 1-year survival was not affected^91^. Overweight (BMI: 25 – 30) recipients had a smaller, but still significantly increased risk of mortality compared with normal weight subjects. Consistent with the potential mechanical consequences of obesity is the finding of an increased likelihood of death from respiratory failure, as well as infection and cardiac disease^91^.

An increased incidence of primary graft dysfunction was detected with obesity and higher plasma leptin levels, suggesting a link between adipose tissue and inflammation^92^. In a subsequent study of the post-LAS only era (after May, 2005), the 1-yr mortality of recipients with a BMI of 30-35 was similar to normal weight individuals, whereas greater degrees of obesity were associated with nearly twice the risk of death^93^. These investigators also found higher mortality with elevated plasma leptin levels among recipients who did not undergo cardiopulmonary bypass. Plasma leptin levels were only weakly related to BMI, but more strongly correlated with percent body fat. The lack of an association with mild obesity and 1-yr mortality was attributed in part to a poor correlation between adiposity, as measured by DXA scanning and BMI. Obesity defined by DXA (>30% body fat in women and > 25% in men) was present in 70% of patients with a BMI < 30. Thus any adverse impact of excess body fat per se would be diluted across the BMI categories. The mechanical consequences of mild obesity may have been attenuated compared with previous studies by better surgical techniques and peri-operative management.

At the other end of the body weight spectrum, reduced BMI (< 18.5) was associated with a 35% increased risk of 1-yr mortality after adjusting for other variables^93^. In the older UNOS analysis, underweight COPD patients had a 64% higher risk of death at 1 year compared with normal weight, but no impact was observed for 5-yr mortality conditional upon 1-yr survival^91^. In contrast, underweight CF recipients had a similar 1-yr survival, but an odds ratio for death at 5 years of 1.58 compared with the normal BMI CF group. On the other hand, a recent single center study of 453 patients found no impact of low BMI, which was present in 11% of the cohort^94^. This report did identify low serum albumin (< 3 gm/dl) as a risk factor for death after transplant. Similarly, an analysis of the UNOS registry demonstrated a 1-yr mortality rate ratio of 1.48 for each 0.5 gm/dl decrease in serum albumin concentration^95^. This effect was strongest for CF recipients, in whom the ratio was 2.28. Reduced serum albumin likely reflects malnutrition as well as systemic inflammation and resultant sarcopenia. Whether intervention with oral supplements or enteral tube feeding prior to transplant can improve nutritional status and reduce transplant risk is not clear. There are no randomized controlled trial data, but observational studies in CF adults suggest that enteral tube feeding leads to weight gain and possible stabilization of lung function in malnourished patients^96^. In cachectic COPD subjects, recent controlled trials, mainly utilizing oral supplementation, have demonstrated significant weight gain, often associated with improvements in respiratory muscle strength and exercise capacity^97^.

#### Coronary artery disease (CAD)

Concomitant CAD is very common among LTX candidates with a prevalence of 60% in one series^98^. The presence of asymptomatic mild-moderate CAD, defined as single or multi-vessel disease with less than 70% stenoses (< 50% if left main coronary artery stenosis) does not appear to have a significant impact on either short- or long-term outcomes^99^. A review of the Duke University experience in 177 patients found no difference in peri-operative cardiac events, duration of ventilation or ICU stay or mortality compared to recipients with no CAD. Revascularization was required in only 6%, all late post-transplant. Severe CAD amenable to revascularization can be managed by either pre-operative percutaneous coronary intervention (PCI) or bypass grafting at the time of transplant. If feasible, bare metal stents are preferred for PCI to minimize the duration of dual anti-platelet therapy required. Clopidogrel must be discontinued prior to listing. Overall, outcomes among recipients requiring revascularization in a series from Duke were similar to controls. However, concomitant bypass surgery was associated with a longer hospital stay and duration of mechanical ventilation^100^.

#### Previous thoracic surgery

Pleural adhesions from prior cardiothoracic surgery can complicate the transplant procedure. The University of Pittsburgh group, where over 40% of recipients had a history of previous cardiothoracic procedures, analyzed their experience with such patients^101^. Simple chest drain placement did not appear to impact outcomes. Pleurodesis, on the other hand, was associated with a 14% rate of re-explorations for bleeding compared with 5% in recipients without a history or cardiothoracic procedures. A significant increase in other adverse outcomes, including phrenic nerve injury, prolonged ventilation and dialysis requirement was also observed. Complication rates among patients with a history of thoracic or cardiac surgery were intermediate between those seen with pleurodesis and tube thoracostomy alone. In a multivariate analysis, chemical pleurodesis, massive transfusion and prolonged cardiopulmonary bypass were associated with increased mortality^101^. Importantly, pre-operative computed tomography reliably predicted surgical difficulties due adhesions in the pleurodesis patients. Anecdotally, mechanical pleurodesis, achieved with dry gauze abrasion during video-assisted thorascopic surgery, appears to induce less pleural adhesions and is preferred over chemical methods if future lung transplant is a possibility. Thus, while previous cardiothoracic surgery, particularly pleurodesis, may increase transplant risk, it is not considered an absolute contraindication^102^. A notable exception is a parietal pleurectomy due the dense adhesions induced by this procedure.

#### Mechanical ventilation and extra-corporeal support

Invasive mechanical ventilation has long been recognized as a strong risk factor for 1-yr mortality post-transplant with a hazard ratio of 1.47 in the ISHLT registry^31^. An analysis of the UNOS database from 1987 – 2008 identified 586 recipients on mechanical ventilation at the time of transplant^103^. One year survival was 62% compared with 79% among non-supported patients. This difference persisted after propensity matching yielding a hazard ratio of 1.49. Similar results were obtained in a more recent study restricted to the post-LAS era where one year survival among 419 ventilated recipients was 68% vs. 80% in propensity matched non-ventilated subjects^104^. Importantly, 3-year survival was similar in the two groups (56% vs. 60%) and the increased risk of death was restricted to the first 6 months being 2-fold higher. Survival among ventilated recipients who survived the first 6 months was not different. Interestingly, there was no impact of mechanical ventilation on survival among COPD patients. Factors associated with 1-yr survival were younger age, better renal function, non-fibrotic lung disease and bilateral lung transplantation^104^.

Thus, while mechanical ventilation is clearly associated with increased post-transplant mortality, the risk is not prohibitive and should be factored into the entire assessment. A key point is that such patients should already be on a waiting list or have nearly completed their evaluation. Emergent consideration of a ventilated patient who has not met the transplant team previously is fraught with enormous difficulties. It is very challenging under such circumstances to adequately ascertain a patient’s candidacy, particularly, from a psychosocial perspective. The impact of the duration of ventilation prior to transplantation has not been investigated. A short period of support prior to transplant in a patient already listed is generally acceptable. However, deconditioning, respiratory muscle weakness and infections are likely to complicate prolonged mechanical ventilation. It is crucial that such patients participate in aggressive physical therapy. A ventilated, non-ambulatory patient is unlikely to survive transplantation. Early tracheotomy and minimization of sedation will facilitate exercise. Whether the need for non-invasive ventilation increases the risk of transplantation is not clear.

Technologic advances in extra-corporeal membrane oxygenation (ECMO) equipment have revolutionized the use of this modality for refractory respiratory failure. ECMO has been applied with increasing frequency in recent years as a bridge to lung transplantation^105^. In the U.S. in 2013, 3.1% of lung transplant recipients were on combined ventilation and ECMO, 1.7% on ECMO only and 5.2% on a ventilator only^19^. This compares with 0.6%, 0% and 1.7%, respectively in 2003. One-year post-transplant survival rates have steadily improved over time from 47% during 2006-08 to 74% during 2009-11^106^. This dramatic improvement in outcomes is likely due to several factors, including technical enhancements and medical team experience^107^. Aggressive physical therapy and early mobility are critical to successful bridging to transplant. A key advantage of ECMO vs. mechanical ventilation is the requirement for less or no sedation. “Awake” ECMO allows for ambulation and rehabilitation, which is felt to contribute to the superior post-transplant outcomes relative to mechanical ventilation^108^. Nevertheless, ECMO use clearly carries an increased risk. Roughly one quarter of candidates supported with ECMO do not survive to lung transplant^108–110^. Those successfully bridged have an increased mortality. In a recent UNOS review, the hazard ratio for death was 1.85 among 198 recipients transplanted off of ECMO support since 2000^111^. The greatest risk was demonstrated among patients ≥ 60 years of age, whereas no increased risk was found for those < 40 years.

## Conclusion

Lung transplantation is an important and increasingly viable option for patients with a variety of end-stage lung diseases. Recipient selection criteria have been expanded in recent years. As outcomes continue to improve and the donor pool expanded, these are likely to undergo further liberalization. Transplant clinicians must make a careful risk-benefit assessment for each individual patient. Potential recipients must undergo a detailed educational process to ensure that they understand all the potential risks and expectations. Early referral is crucial to allow sufficient time for a comprehensive, multi-disciplinary evaluation and optimization of modifiable factors (e.g. deconditioning, obesity).
